# Minimal important difference, patient acceptable symptom state and longitudinal validity of oxford elbow score and the quickDASH in patients with tennis elbow

**DOI:** 10.1186/s12874-023-01934-4

**Published:** 2023-07-06

**Authors:** Teemu Karjalainen, Tuomas Lähdeoja, Mikko Salmela, Clare L Ardern, Joona Juurakko, Teppo LN Järvinen, Simo Taimela, FINITE investigators

**Affiliations:** 1 Department of Hand and Microsurgery, Tampere University Hospital, Tampere, Finland; 2 Central Finland Healthcare District, Hospital Nova, Hoitajantie 3, Jyväskylä, 40620 Finland; 3 Finnish Centre for Evidence-Based Orthopaedics (FICEBO), Department of Orthopaedics and Traumatology, University of Helsinki and Helsinki University Hospital, Helsinki, Finland

**Keywords:** Clinimetrics, DASH, Oxford elbow score, Tennis elbow, Minimal clinically important change, MID, MCID, Patient accepted symptom state, PASS

## Abstract

**Background:**

The Oxford Elbow Score (OES) and the short version of Disabilities of Arms, Shoulder and Hand (QuickDASH) are common patient-reported outcomes for people with elbow problems. Our primary objective was to define thresholds for the Minimal Important Difference (MID) and Patient-Acceptable Symptom State (PASS) for the OES and QuickDASH. The secondary aim was to compare the longitudinal validity of these outcome measures.

**Methods:**

We recruited 97 patients with clinically-diagnosed tennis elbow for a prospective observational cohort study in a pragmatic clinical setting. Fifty-five participants received no specific intervention, 14 underwent surgery (11 as primary treatment and 4 during follow-up), and 28 received either botulinum toxin injection or platelet rich plasma injection. We collected OES (0 to 100, higher is better) and QuickDASH (0 to 100, higher is worse), and global rating of change (as an external transition anchor question) at six weeks, three months, six months and 12 months. We defined MID and PASS values using three approaches. To assess the longitudinal validity of the measures, we calculated the Spearman’s correlation coefficient between the change in the outcome scores and external transition anchor question, and the Area Under the Curve (AUC) from a receiver operating characteristics (ROC) analysis. To assess signal-to-noise ratio, we calculated standardized response means.

**Results:**

Depending on the method, MID values ranged from 16 to 21 for OES Pain; 10 to 17 for OES Function; 14 to 28 for OES Social-psychological; 14 to 20 for OES Total score, and − 7 to -9 for QuickDASH. Patient-Acceptable Symptom State (PASS) cut offs were 74 to 84 for OES Pain; 88 to 91 for OES Function; 75 to 78 with OES Social-psychological; 80 to 81 with OES Total score and 19 to 23 with Quick-DASH. OES had stronger correlations with the anchor items, and AUC values suggested superior discrimination (between improved and not improved) compared with QuickDASH. OES also had superior signal-to-noise ratio compared with QuickDASH.

**Conclusion:**

The study provides MID and PASS values for OES and QuickDASH. Due to better longitudinal validity, OES may be a better choice for clinical trials.

**Trial registration:**

ClinicalTrials.gov NCT02425982 (first registered April 24, 2015).

**Supplementary Information:**

The online version contains supplementary material available at 10.1186/s12874-023-01934-4.

## Introduction

Elbow-specific Oxford Elbow Score (OES), and the short version of the upper limb-specific Disabilities of Arms, Shoulder and Hand (QuickDASH) both use a question-and-response format to convert a complex multidimensional health state into a single metric scale [1, 2]. While a single score facilitates comparisons of outcomes in a trial, it can also be a source of confusion: How to easily integrate a metric score into decision-making in clinical practice, if patients are not familiar with the measure?

Two established concepts help clinicians and patients interpret PROMs: Minimal (clinically) important difference (M(C)ID) and Patient Accepted Symptom State (PASS) [3, 4]. MID represents “*the smallest difference in score in the outcome of interest that informed patients or informed proxies perceive as important, either beneficial or harmful, and that would lead the patient or clinician to consider a change in the managemen*t” [3]. PASS represents the value of the score beyond which patients consider themselves well [4]. While MID can be used to interpret if the mean difference between groups is relevant [5] or how large proportion of participants experience meaningful change (responder analysis), PASS can only be used for the latter purpose.

The OES was developed to measure outcomes of elbow surgery [2]. Since the items specifically address limitations due to elbow problems, it is likely well suited to measure outcomes in people with tennis elbow. The OES consists of three subscales: pain, function and social-psychological scale. The QuickDASH is a common upper extremity-specific PROM. The published MIDs for QuickDASH and OES are not specific for people with tennis elbow [6–9]. It is also unclear whether the OES or the QuickDASH is better suited to measuring change in the health state (i.e. which measure has better longitudinal validity), and we are not aware of studies estimating PASS values for the Oxford Elbow Score.

The primary objective of this study was to estimate MID and PASS values for OES and QuickDASH in people with tennis elbow to facilitate interpretation of future trials and meta-analyses. The secondary aim was to assess the longitudinal validity of these measures to see which outcome was more responsive to change in the health state. A more responsive outcome is more efficient in trials. We used data from a longitudinal/prospective pragmatic observational cohort study involving patients who received different treatments for tennis elbow.

## Methods

The Helsinki University Hospital institutional review board approved the study protocol. Recruitment began in May 2015 and finished in March 2018.

### Design and setting

Participants were referred to one of six study centres (orthopaedic or hand surgery outpatient clinics) in Finland with tennis elbow that was not responsive to usual care.

### Participants

All patients were screened for eligibility by a hand surgeon or an upper extremity-focussed specialist orthopaedic surgeon at each centre. The diagnosis was based on history and clinical examination. The screening and recruitment protocol did not require any imaging, but imaging findings were considered if they were available.

The inclusion criteria for the cohort study were:

- Clinical diagnosis of tennis elbow (pain on the lateral side of the elbow, made worse by pressure applied on the lateral epicondyle of the humerus and during resisted extension of the wrist or when making a tight fist with a straight elbow joint),
- Symptom duration of over 10 months,
- Age between 35 and 60 years,
- Ability to read and comprehend the questionnaires,
- Provided informed consent.

The exclusion criteria were:

- Inflammatory or neurological condition (including nerve entrapment) affecting upper limb function,
- Signs of instability of the elbow joint (table top or lateral pivot shift test),
- Radiographic elbow osteoarthritis,
- Pain to the distal biceps tendon or the medial side of the elbow indicative of biceps tendinopathy or medial epicondylitis,
- Previous surgery, fracture or dislocation of the elbow,
- Congenital deformity in the elbow region,
- Systemic muscle, tendon, nerve or joint disease,
- Painful snapping or crepitus of the elbow joint,
- A limitation of passive range of motion of the elbow > 10 degrees,
- Abnormal elbow radiograph.

Before enrolment, participants were informed about the study and provided written informed consent. Participants were not taking part in any other study of tennis elbow.

### Baseline data and outcome measures

We collected the following information at the baseline during the initial visit at the clinic: Age, sex, duration of symptoms, previous corticosteroid injections, symptomatic side, history of smoking, OES and QuickDASH.

The OES subscales (pain, function, and social-psychological) comprise four questions that are weighed equally [2]. The subscales were originally intended to be used separately, but they can be summed to a total score. The score (each subscale or total) can be converted to a metric scale (0 to 100 points, higher score indicating better outcome).

QuickDASH comprises 10 questions about pain and disability affecting upper limbs in daily activities. It is a short form of DASH questionnaire (30 questions) with comparable clinimetric properties as the DASH [1]. QuickDASH scores are from 0 to 100; higher score indicating worse outcome.

We used Global Rating of Change (GRC) as an external transition anchor question (to define MID and assess longitudinal validity). Participants rated the overall change in health state compared to the baseline measure in a 6-step Likert scale (much worse-little worse-unchanged-little better-much better-complete recovery). For state (to define PASS), we asked the participants whether they would be satisfied with the current *state* of the global elbow function (yes/no).

### Interventions

All participants were informed about the benign course of the condition. They were primarily offered wait and see strategy, but if they wanted active treatment, they were offered botulinum toxin injection, platelet rich plasma injection or surgery, depending on the centre and patient. Shared decision making was used to decide the course of treatment; the study protocol excluded only further corticosteroid injections.

### Data collection and analysis

We collected the outcomes at baseline, six weeks, three months, six months, and 12 months. We calculated the change in OES and QuickDASH scores for each patient by subtracting baseline score from the score at the follow-up point. A positive change score indicates improvement in OES and worsening in the QuickDASH score. Due to small number of participants reporting little better or no change at some time points, we combined all time points (i.e. all transition anchor – target measures pairs were combined in a single analysis).

To assess the longitudinal validity and credibility of MID values, we calculated the Spearman’s correlations between GRC and (1) change scores; (2) absolute scores; and (3) baseline score. Moderate to high (> 0.5) correlation between the anchor and change score suggests validity while values < 0.4 suggest low validity of the anchor.^4^ We also assessed the Area Under Curve (AUC) -values from the receiver operating characteristics (ROC) analysis to see how well the score could discriminate those who reported feeling better compared with those who remained unchanged.

We determined the MID using three different anchor-based methods: **(1)** Mean change method (MID = mean change of participants reporting ‘little better’) **(2)** Mean difference of change method (MID = [mean change of ‘little better’] – [mean change of ‘unchanged’]) **(3)** ROC curve method (closest point to the upper left corner) dichotomizing between ‘no change’ and ‘little better’ and excluding participants with responses ‘little worse’ or ‘much worse’ [10].

For PASS, we used 75th percentile method and ROC method from the 6-month and 12-month time points [4]. In the 75th percentile method, PASS was defined as the 25th percentile score for OES (i.e. 75% of participants who considered themselves well had a score above this cut off) and 75th percentile score for QuickDASH (i.e. 75% of the participants who considered themselves well had a score under this cut off). The ROC method was used to find the optimal cut off discriminating between the participants who reported acceptable state and those not who did not.

To assess the signal-to-noise ratio, we calculated standardized response mean (SRM) for both outcomes as mean score change (between baseline and 1 year follow-up) divided by the SD of the change. SRM is the ratio of signal to noise. A SRM of 0.2 can be considered to indicate low responsiveness; 0.5 moderate and 0.8 as large responsiveness [11].

## Results

We recruited 97 participants with clinically diagnosed tennis elbow (Table 1). At baseline, OES and QuickDASH data were available for 93 and 91 participants, respectively (4 and 6 missing due to too many missing items). Seventy-four (76%) participants returned the outcome questionnaires at six weeks, 72 (74%) at three months, 74 (76%) at six months and 75 (77%) at 12 months.

Most (n = 55; 61%) participants did not receive any specific intervention initially; four participants received surgery during follow-up. Smoking was fairly common (27%) among the participants, and most (71%) had received at least one corticosteroid injection before enrolment (range 1 to 6 injections) (Table 1).

**Table 1:** Characteristic of the participants (n = 97) and treatments received

Characteristic	Mean (SD) or n (%)
Age, mean (SD)	49 (5.3)
Duration of symptoms, months	19 (12)
Sex, Female (%)	51 (53)
Symptomatic side, right (%)	67 (69)
Smoker (%)	26 (27)
Previous corticosteroid injection	69 (71)
Initial treatment in this study	
No treatment	59 (61)
Surgery	11 (11)
PRP injection	13 (13)
Botulinus injection	14 (14)

SD = standard deviation, n = number, PRP = platelet-rich plasma

Most participants improved during the follow-up (Additional file 1). Both QuickDASH and OES captured the perceived change adequately, but OES change correlated more strongly with the transition anchor compared with QuickDASH change (Additional file 1), and accordingly, OES discriminated better (Table 2). Correlations were: OES Total − 0.76 (95% CI -0.80 to -0.72); OES pain − 0.75 (95% CI -0.80 to -0.71); OES function − 0.64 (95% CI -0.70 to -0.58); OES psycho-social − 0.7 (95% CI -0.75 to -0.66) and QuickDASH 0.57 (95% CI 0.50 to 0.65). The OES and its subscales also had better signal-to-noise ratio (standardized response mean) compared with QuickDASH (Table 3).

**Table 2:** MID estimates, sensitivity and specificity of the cut offs, and AUC-values from the ROC analysis

Outcome measure	MID	Sensitivity	Specificity	AUC (95% CI)
OES Pain	16	0.82	0.8	0.89 (0.85 to 0.92)
OES Function	16	0.74	0.71	0.81 (0.76 to 0.86)
OES Social-Psychological	28	0.71	0.85	0.85 (0.81 to 0.89)
OES Total	20	0.79	0.86	0.88 (0.85 to 0.92)
QuickDASH	-9	0.71	0.75	0.78 (0.73 to 0.83)

*OES scaled 0 to 100 (higher is better), QuickDASH scaled 0 to 100 (higher is worse).*

MID = minimal clinically important difference, AUC = Area Under the Curve, CI = Confidence interval, ROC = receiver operating characteristics, OES = The Oxford Elbow Score, DASH = Disabilities of Arms, Shoulder and Hand

**Table 3:** Standardized response means for different measures at each time point

	OES Pain (95% CI)	OES Function (95% CI)	OES social-psychosocial (95% CI)	OES total (95% CI)	QuickDASH (95% CI)
6 weeks	0.51 (0.27 to 0.75)	0.40 (0.17 to 0.64)	0.62 (0.38 to 0.86)	0.61 (0.37 to 0.85)	0.21 (0.03 to 0.44)
3 months	0.82 (0.59 to 1.06)	0.81 (0.57 to 1.05)	0.96 (0.73 to 1.20)	0.96 (0.73 to 1.20)	0.66 (0.42 to 0.90)
6 months	1.05 (0.81 to 1.28)	0.96 (0.73 to 1.19)	1.34 (1.11 to 1.58)	1.20 (0.96 to 1.44)	0.85 (0.62 to 1.09)
12 months	1.26 (1.03 to 1.50)	1.16 (0.93 to 1.39)	1.61 (1.38 to 1.84)	1.43 (1.19 to 1.66)	0.79 (0.55 to 1.03)

OES = The Oxford Elbow Score, CI = Confidence interval, DASH = Disabilities of Arms, Shoulder and Hand

For both OES and QuickDASH change scores, the correlations with the transition anchor (GRC) increased at longer follow-up. After three months, the change scores correlated more strongly with the current state than with change score (Additional file 1). The change scores correlated with the baseline score only for OES at 24 months (Additional file 1).

MID = minimal clinically important difference, ROC = receiver operating characteristics, OES = The Oxford Elbow Score, DASH = Disabilities of Arms, Shoulder and Hand, CI = Confidence interval.

MID and PASS estimates varied depending on the calculation method (Tables 4 and 5). The PASS values fell between 10 and 25 points (or 10–25% of the scale) from the best possible score (Table 5).

## Discussion

In people with tennis elbow, the elbow-specific OES better captured the change in the perceived health than did the upper extremity-specific QuickDASH. The OES better discriminated people who improved from people who reported no change, and the MID and PASS values are therefore more credible for OES than QuickDASH. OES also had superior signal-to-noise ratio, indicating that smaller sample sizes may be used in clinical trials to identify or exclude clinically-relevant differences when OES is used compared to QuickDASH.

The MID reflects the difference in score that informed patients perceive as important (either beneficial or harmful), and that would lead the patient or clinician to consider a change in management. The methods impacted the MID values adding a layer of uncertainty to the interpretation of the results. If we determine the MID using the mean change in people reporting being ‘little better’, approximately half of the participants who reported feeling better will be classified as ‘not improved’— a relatively high rate of misclassification. ROC analysis optimizes the cut off, therefore MID from ROC analysis is more valid for discrimination purposes. Due to the impact of outliers, the ROC method is also more robust to skewed distributions compared to the mean change method or the mean difference of change method. However, ROC analysis is affected by the prevalence of people who report improvement. With larger prevalence, the MID values tend to be larger [12]. There is no consensus about the optimal method, but a recent credibility instrument can be used assess the credibility of the MID estimates [13].

**Table 4:** MID estimates

Outcome measure	Mean difference of change^a^	Mean change^b^	ROC
OES Pain	17 (12 to 22)	21 (18 to 25)	16
OES Function	10 (5 to 15)	17 (13 to 21)	16
OES Social-Psychological	14 (8 to 20)	24 (20 to 29)	28
OES Total	14 (9 to 18)	21 (17 to 24)	20
QuickDASH	-7 (-13 to -1)	-9 (-13 to -4)	-9

*Values are MID with 95% CI except ROC which have only MID values. OES scaled 0 to 100 (higher is better), QuickDASH scaled 0 to 100 (higher is worse)*

^*a*^ *Mean difference of change = Mean difference between those who reported little better and no change*

^*b*^ *Mean change = Mean change in participants who reported little better*

**Table 5:** Patient Acceptable Symptom State (PASS) estimates

Outcome measure	75th percentile method	ROC method			
	PASS	PASS	Sensitivity	Specificity	AUC (95% CI)
OES Pain	75	84	0.68	0.87	0.85 (0.81 to 0.89)
OES Function	88	91	0.72	0.88	0.83 (0.78 to 0.87)
OES Social-Psychological	75	78	0.73	0.88	0.88 (0.84 to 0.91)
OES Total	81	80	0.76	0.86	0.88 (0.84 to 0.91)
QuickDASH	23	19	0.69	0.77	0.80 (0.75 to 0.85)

*OES scaled 0 to 100 (higher is better), QuickDASH scaled 0 to 100 (higher is worse)*

ROC = receiver operating characteristics, AUC = Area Under the Curve, OES = The Oxford Elbow Score, DASH = Disabilities of Arms, Shoulder and Hand

We propose that differences that are smaller than the lowest values from our study (16 for OES pain; 10 for OES function; 14 for OES psycho-social; 14 for OES pain; and 7 of quickDASH) are judged as not meaningful in people with tennis elbow. For example, authors of a trial or meta-analysis should conclude that the intervention does not provide clinically-important benefits only if the treatment effect confidence intervals are below the smallest provided values. Conversely, higher differences than the highest values (21 for OES pain; 17 for OES function; 28 for OES social-psychological; and 9 for QuickDASH) should be considered indicating relevant benefits. When the difference is within the range of possible MID or PASS values, some uncertainty regarding the conclusions is appropriate.

Our MID estimates correspond reasonably well with the previous studies. The developers of the OES found MID values of 9, 18, and 18 points with pain, function, and social-psychological scale respectively in a heterogenous population undergoing surgery for elbow complaints. One in ten patients in their sample received surgery for tennis elbow [6]. Another study found lower MID values, 7 points for OES pain, 6 points for OES function, 12 points for OES social-psychological and 8 points for OES total score in people with simple elbow dislocation [9]. This demonstrates how the sample and condition can affect the MID estimates. Further, our MID values for QuickDASH also correspond well with previous studies in various elbow and shoulder conditions [7–9]. Recently, a consensus paper recommended PRTEE for core outcome set for disability domain in people with tennis elbow [14]. Although the recommendation did not include OES or quickDASH, both quickDASH and OES both scored well in EMPRO scores supporting our findings.

Patient accepted symptom state is a cut off for PROM beyond which people consider being well [4]. In our sample, the cut off was approximately 10–25 points (%) worse than the best possible score. PASS values can be used to calculate the proportion of people achieving satisfactory state in responder analyses if such comparison is considered appropriate to interpret the effect (e.g. NNT calculation). PASS may be a more valid cut off than a change of at least the MID because achieving PASS is not as dependent on the baseline symptom burden [4, 15] – an improvement of MID can be largely meaningless in patients with high baseline symptom burden, whereas starting with a low value, one can reach complete recovery. Furthermore, due to risks and high costs related to surgery, reaching PASS may be a more appropriate treatment objective in surgical trials compared with MID – the smallest improvement patients can perceive may not be sufficient objective for surgery.

Recall bias affected the patient global improvement assessment beyond three months. When participants were asked to report whether they were better compared to baseline, their responses were more closely associated with their current status than how their condition had changed. This is typical with musculoskeletal conditions [16]. Clinicians and researchers must consider this when interpreting global transition measures. Another limitation was the follow-up rate of just under 80% at every time point, which introduces some uncertainty to the estimates as it is not plausible that the data were missing completely at random. Also, the results are applicable only to the study’s instruments. Despite the similarities, the results may not generalise to the full DASH, and a recent Delphi consensus study did not include the OES or quickDASH in a core outcome set for this population [14]. The strength of our study is the high correlation between the anchor and target instrument providing credible MID estimates that discriminate well people who experienced meaningful improvement from those who had not.

## Conclusion

This study determined credible MID and PASS values that can be used to interpret trial results. Comparison of longitudinal validity of OES and QuickDASH in tennis elbow patients suggests that although the performance of QuickDASH is acceptable, OES has better signal-to-noise ratio and better longitudinal validity. The OES may be better option as an outcome measure in clinical trials.

## Electronic supplementary material

Below is the link to the electronic supplementary material.

Supplementary Material 1

## Data Availability

The datasets generated and/or analysed during the current study are not publicly available due to data privacy regulations, but are available from the corresponding author on reasonable request.
