# Supplementary material for: Minimal important difference, patient acceptable symptom state and longitudinal validity of oxford elbow score and the quickDASH in patients with tennis elbow
Source: BMC Med Res Methodol. 2023 Jul 6;23:158. doi: 10.1186/s12874-023-01934-4 (PMC10324132; doi:10.1186/s12874-023-01934-4)
Supplement: Supplementary file 1 — Supplementary Material 1 [file 12874_2023_1934_MOESM1_ESM.docx]

**Additional File 1**

**Table S1. OES and QuickDASH scores changes according to GRC categories**

|  | **Baseline** | | |  | **GRC at 6 weeks** | | | **GRC at 3 months** | | | **GRC at 6 months** | | | **GRC at 12 months** | | |
| --- | --- | --- | --- | --- | --- | --- | --- | --- | --- | --- | --- | --- | --- | --- | --- | --- |
|  | **N** | **Mean** | **SD** |  | **N** | **Mean change** | **SD** | **N** | **Mean change** | **SD** | **N** | **Mean change** | **SD** | **N** | **Mean change** | **SD** |
| OES pain | 93 | 43.8 | 18.2 | Completely recovered | 0 | n/a | n/a | 0 | n/a | n/a | 3 | 52.1 | 13.0 | 10 | 56.9 | 17.8 |
|  |  |  |  | Much better | 6 | 32.3 | 14.5 | 21 | 33.9 | 18.6 | 34 | 42.5 | 25.8 | 36 | 45.5 | 24.4 |
|  |  |  |  | Somewhat better | 23 | 20.1 | 15.2 | 20 | 18.1 | 2.1 | 15 | 25.4 | 15.0 | 16 | 23.8 | 20.4 |
|  |  |  |  | Unchanged | 36 | 3.8 | 13.1 | 23 | 7.9 | 2.1 | 15 | 3.8 | 18.6 | 5 | -2.5 | 15.7 |
|  |  |  |  | Somewhat worse | 4 | 3.1 | 3.6 | 4 | 0.0 | 2.0 | 4 | 0.0 | 11.4 | 4 | 4.7 | 11.8 |
|  |  |  |  | Much worse | 4 | -21.9 | 25.8 | 2 | -34.4 | 3.5 | 1 | 6.3 | n/a | 2 | -6.3 | 0.0 |
| OES function | 93 | 60.0 | 18.0 | Completely recovered | 0 | n/a | n/a | 0 | n/a | n/a | 3 | 27.1 | 15.7 | 10 | 39.4 | 17.9 |
|  |  |  |  | Much better | 6 | 18.8 | 19.8 | 21 | 31.5 | 3.5 | 34 | 36.8 | 21.6 | 37 | 38.3 | 23.6 |
|  |  |  |  | Somewhat better | 23 | 11.7 | 21.3 | 20 | 16.9 | 2.7 | 16 | 20.3 | 16.8 | 16 | 19.1 | 16.5 |
|  |  |  |  | Unchanged | 36 | 6.3 | 14.2 | 23 | 12.8 | 2.6 | 15 | 2.5 | 15.3 | 5 | 0.0 | 25.8 |
|  |  |  |  | Somewhat worse | 4 | 3.1 | 12.0 | 4 | -1.6 | 2.8 | 4 | 3.1 | 10.8 | 4 | 7.8 | 7.9 |
|  |  |  |  | Much worse | 4 | -17.2 | 12.9 | 2 | -31.3 | 1.4 | 1 | -31.3 | n/a | 2 | -9.4 | 22.1 |
| OES psychosocial function | 93 | 36.4 | 19.5 | Completely recovered | 0 | n/a | n/a | 0 | n/a | n/a | 3 | 37.5 | 6.3 | 10 | 53.1 | 17.2 |
|  |  |  |  | Much better | 6 | 40.6 | 14.7 | 21 | 33.9 | 3.7 | 34 | 47.8 | 18.8 | 37 | 54.7 | 22.0 |
|  |  |  |  | Somewhat better | 22 | 15.1 | 15.9 | 20 | 19.4 | 2.9 | 15 | 32.9 | 18.7 | 16 | 34.8 | 18.7 |
|  |  |  |  | Unchanged | 36 | 9.2 | 21.0 | 23 | 13.0 | 2.2 | 15 | 11.7 | 20.6 | 5 | -5.0 | 20.0 |
|  |  |  |  | Somewhat worse | 4 | 9.4 | 10.8 | 4 | 3.1 | 2.5 | 4 | 7.8 | 21.3 | 4 | 14.1 | 15.6 |
|  |  |  |  | Much worse | 4 | -3.1 | 23.1 | 2 | -3.1 | 4.9 | 1 | -25.0 | n/a | 2 | 12.5 | 2.8 |
| OES total | 93 | 46.8 | 16.1 | Completely recovered | 0 | n/a | n/a | 0 | n/a | n/a | 3 | 38.9 | 9.4 | 10 | 49.8 | 15.1 |
|  |  |  |  | Much better | 6 | 30.6 | 14.2 | 21 | 33.1 | 18.9 | 34 | 42.3 | 19.9 | 36 | 46.2 | 21.7 |
|  |  |  |  | Somewhat better | 22 | 15.4 | 14.2 | 20 | 18.1 | 12.4 | 14 | 26.9 | 15.2 | 16 | 25.9 | 16.2 |
|  |  |  |  | Unchanged | 36 | 6.4 | 13.3 | 23 | 11.2 | 12.5 | 15 | 6.0 | 16.4 | 5 | -2.5 | 18.9 |
|  |  |  |  | Somewhat worse | 4 | 5.2 | 6.5 | 4 | 0.5 | 12.3 | 4 | 3.6 | 11.5 | 4 | 8.9 | 7.5 |
|  |  |  |  | Much worse | 4 | -14.1 | 20.0 | 2 | -22.9 | 20.6 | 1 | -16.7 | n/a | 2 | -1.0 | 13.3 |
| DASH change | 91 | 41.7 | 20.1 | Completely recovered | 0 | n/a | n/a | 0 | n/a | n/a | 3 | -28.4 | 9.7 | 9 | -29.8 | 11.1 |
|  |  |  |  | Much better | 5 | -21.3 | 19.3 | 21 | -21.9 | 18.1 | 34 | -25.8 | 20.9 | 36 | -31.0 | 24.7 |
|  |  |  |  | Somewhat better | 22 | -7.6 | 18.1 | 19 | -4.6 | 15.1 | 15 | -19.5 | 20.6 | 15 | -5.4 | 24.8 |
|  |  |  |  | Unchanged | 35 | 0.2 | 15.3 | 22 | -9.2 | 11.0 | 15 | 1.0 | 12.3 | 4 | 10.3 | 32.6 |
|  |  |  |  | Somewhat worse | 4 | -1.0 | 14.7 | 4 | -7.4 | 11.9 | 3 | -8.5 | 17.7 | 4 | -16.5 | 11.4 |
|  |  |  |  | Much worse | 4 | 5.3 | 18.3 | 2 | 16.4 | 10.3 | 1 | -13.9 | n/a | 2 | 7.5 | 28.3 |

OES = The Oxford Elbow Score, DASH = Disabilities of Arms, Shoulder and Hand, GRC = Global Rating of Change, N = number, SD = standard deviation, n/a = not applicable

**Table S2. Correlations between global rating of change and the change of the target instrument**

| **Time points** | **OES Pain** | **OES Function** | **OES Psychosocial** | **OES Total** | **QuickDASH** |
| --- | --- | --- | --- | --- | --- |
| 6 weeks | -0.64^***^ (-0.79 to -0.52) | -0.33^**^ (-0.56 to -0.14) | -0.36^**^ (-0.60 to -0.18) | -0.49^***^ (-0.70 to -0.33) | 0.30^*^ (0.08 to 0.56) |
| 3 months | -0.66^***^ (-0.81 to -0.54) | -0.47^***^ (-0.69 to -0.30) | -0.44^***^ (-0.65 to -0.27) | -0.58^***^ (-0.77 to -0.44) | 0.34^**^ (0.13 to 0.59) |
| 6 months | -0.64^***^ (-0.79 to -0.52) | -0.62^***^ (-0.79 to -0.5) | -0.62^***^ (-0.80 to -0.48) | -0.69^***^ (-0.84 to -0.58) | 0.49^***^ (0.33 to 0.70) |
| 12 months | -0.66^***^ (-0.8 to -0.54) | -0.54^***^ (-0.71 to -0.39) | -0.55^***^ (-0.74 to -0.41) | -0.64^***^ (-0.78 to -0.53) | 0.46^***^ (0.32 to 0.64) |
| 24 months | -0.73^***^ (-0.87 to -0.63) | -0.65^***^ (-0.81 to -0.52) | -0.67^***^ (-0.82 to -0.56) | -0.74^***^ (-0.88 to -0.65) | 0.63^***^ (0.50 to 0.81) |
| All time points | -0.75^***^ (-0.80 to -0.71) | -0.64^***^ (-0.70 to -0.58) | -0.7^***^ (-0.75 to -0.66) | -0.76^***^ (-0.80 to -0.72) | 0.57^***^ (0.50 to 0.65) |

*Values are Spearman’s rho with 95 % CI. ^*^ p < 0.05. ^**^ p < 0.01. ^***^ p < 0.001.*

OES = The Oxford Elbow Score, DASH = Disabilities of Arms, Shoulder and Hand

**Table S3. Correlations between global rating of change and post scores (state) of the target instrument**

| **Time points** | **OES Pain** | **OES Function** | **OES Psychosocial** | **OES Total** | **QuickDASH** |
| --- | --- | --- | --- | --- | --- |
| 6 weeks | -0.51^***^ (-0.71 to -0.36) | -0.18 (-0.42 to 0.05) | -0.25^*^ (-0.51 to -0.02) | -0.34^**^ (-0.58 to -0.13) | 0.27^*^ (0.05 to 0.52) |
| 3 months | -0.63^***^ (-0.80 to -0.49) | -0.51^***^ (-0.72 to -0.35) | -0.48^***^ (-0.69 to -0.30) | -0.64^***^ (-0.81 to -0.50) | 0.41^***^ (0.20 to 0.64) |
| 6 months | -0.79^***^ (-0.90 to -0.72) | -0.76^***^ (-0.89 to -0.66) | -0.74^***^ (-0.87 to -0.64) | -0.81^***^ (-0.92 to -0.74) | 0.63^***^ (0.50 to 0.80) |
| 12 months | -0.78^***^ (-0.89 to -0.68) | -0.69^***^(-0.84 to -0.59) | -0.72^***^ (-0.86 to -0.62) | -0.78^***^ (-0.90 to -0.70) | 0.58^***^ (0.43 to 0.76) |
| 24 months | -0.78^***^ (-0.92 to -0.69) | -0.68^***^ (-0.85 to -0.56) | -0.8^***^ (-0.89 to -0.72) | -0.82^***^ (-0.92 to -0.75) | 0.66^***^ (0.53 to 0.83) |
| All time points | -0.79^***^ (-0.84 to -0.75) | -0.72^***^ (-0.78 to -0.67) | -0.73^***^ (-0.79 to -0.68) | -0.8^***^ (-0.85 to -0.76) | 0.63^***^ (0.56 to 0.70) |

*Values are Spearman’s rho with 95 % CI. ^*^ p < 0.05. ^**^ p < 0.01. ^***^ p < 0.001.*

OES = The Oxford Elbow Score, DASH = Disabilities of Arms, Shoulder and Hand

**Table S4. Correlations between global rating of change and target instrument baseline scores**

| **Time points** |  | **OES Pain** | **OES Function** | **OES Psychosocial** | **OES Total** | **QuickDASH** |
| --- | --- | --- | --- | --- | --- | --- |
| 6 weeks |  | 0.03 (-0.22 to 0.28) | 0.17 (-0.04 to 0.39) | 0.11 (-0.12 to 0.34) | 0.14 (-0.08 to 0.38) | -0.04 (-0.28 to 0.19) |
| 3 months |  | -0.01 (-0.25 to 0.24) | 0.12 (-0.12 to 0.38) | 0.01 (-0.22 to 0.26) | 0.04 (-0.20 to 0.30) | 0.06 (-0.18 to 0.30) |
| 6 months |  | -0.04 (-0.3 to 0.19) | 0.06 (-0.15 to 0.28) | -0.04 (-0.29 to 0.19) | -0.02 (-0.27 to 0.22) | 0.12 (-0.11 to 0.36) |
| 12 months |  | 0.09 (-0.13 to 0.33) | 0.16 (-0.08 to 0.38) | 0.09 (-0.16 to 0.33) | 0.12 (-0.10 to 0.36) | -0.05 (-0.28 to 0.17) |
| 24 months |  | 0.28* (0.06 to 0.52) | 0.24* ( 0.04 to 0.50) | 0.26* (0.06 to 0.49) | 0.3* (0.10 to 0.54) | -0.15 (-0.40 to 0.09) |

*Values are Spearman’s rho with 95 % CI. ^*^ p < 0.05.*

OES = The Oxford Elbow Score, DASH = Disabilities of Arms, Shoulder and Hand

**Acknowledgements**

FINITE investigators who were part of the consortium and recruited, assessed and treated the participants but did not directly particate in the writing of the manuscript were: Toni Luokkala, Olli-Pekka Kangasniemi, Imke Höfling, Olli Leppänen, Matti Juntunen and Markus Pääkkönen
